# Fluctuations in the incidence of type 1 diabetes in the United States from 2001 to 2015: a longitudinal study

**DOI:** 10.1186/s12916-017-0958-6

**Published:** 2017-11-08

**Authors:** Mary A. M. Rogers, Catherine Kim, Tanima Banerjee, Joyce M. Lee

**Affiliations:** 1 Department of Internal Medicine, University of Michigan, Building 16, Room 422 W North Campus Research Complex, 2800 Plymouth Road, Ann Arbor, Michigan 48109-2800 USA; 2 Institute of Healthcare Policy and Innovation, University of Michigan, Ann Arbor, Michigan USA; 3 Department of Obstetrics & Gynecology, University of Michigan, Ann Arbor, Michigan USA; 4 Department of Epidemiology, University of Michigan, Ann Arbor, Michigan USA; 5 Department of Pediatrics and Communicable Diseases, University of Michigan, Ann Arbor, Michigan USA

**Keywords:** Type 1 diabetes mellitus, Incidence study, Trends, Age differences

## Abstract

**Background:**

While the United States has the largest number of children with type 1 diabetes mellitus, less is known regarding adult-onset disease. The present study utilizes nationwide data to compare the incidence of type 1 diabetes in youth (0–19 years) to that of adults (20–64 years).

**Methods:**

In this longitudinal study, the Clinformatics® Data Mart Database was used, which contains information from 61 million commercially insured Americans (years 2001–2015). Incidence rates and exact Poisson 95% confidence intervals were calculated by age group, sex, census division, and year of diagnosis. Changes in rates over time were assessed by negative binomial regression.

**Results:**

Overall, there were 32,476 individuals who developed type 1 diabetes in the cohort. The incidence rate was greatest in youth aged 10–14 years (45.5 cases/100,000 person-years); however, because adulthood spans over a longer period than childhood, there was a greater number of new cases in adults than in youth (n = 19,174 adults; n = 13,302 youth). Predominance in males was evident by age 10 and persisted throughout adulthood. The male to female incidence rate ratio was 1.32 (95% CI 1.30–1.35). The incidence rate of type 1 diabetes in youth increased by 1.9% annually from 2001 to 2015 (95% CI 1.1–2.7%; *P* < 0.001), but there was variation across regions. The greatest increases were in the East South Central (3.8%/year; 95% CI 2.0–5.6%; *P* < 0.001) and Mountain divisions (3.1%/year; 95% CI 1.6–4.6%; *P* < 0.001). There were also increases in the East North Central (2.7%/year; *P* = 0.010), South Atlantic (2.4%/year; *P* < 0.001), and West North Central divisions (2.4%/year; *P* < 0.001). In adults, however, the incidence decreased from 2001 to 2015 (−1.3%/year; 95% CI −2.3% to −0.4%; *P* = 0.007). Greater percentages of cases were diagnosed in January, July, and August for both youth and adults. The number of new cases of type 1 diabetes (ages 0–64 years) in the United States is estimated at 64,000 annually (27,000 cases in youth and 37,000 cases in adults).

**Conclusions:**

There are more new cases of type 1 diabetes occurring annually in the United States than previously recognized. The increase in incidence rates in youth, but not adults, suggests that the precipitating factors of youth-onset disease may differ from those of adult-onset disease.

**Electronic supplementary material:**

The online version of this article (10.1186/s12916-017-0958-6) contains supplementary material, which is available to authorized users.

## Background

Of any country, the United States has the largest number of children with type 1 diabetes in the world [1]. While there is no reporting system that captures all cases nationwide, there is active surveillance in certain areas of the country, provided by the SEARCH for Diabetes in Youth Study [2], for cases occurring in youth (ages < 20 years). This study provides invaluable information regarding the characteristics of youth with type 1 diabetes, but it does not cover the entire United States nor does it explore adult-onset type 1 diabetes.

Data on disease incidence rates are crucial, providing the underpinnings necessary for etiologic investigations and essential health services. This is of particular importance for type 1 diabetes since its incidence is rising in various countries worldwide [3]. Moreover, while type 1 diabetes has historically been characterized as having an onset during childhood, there has been recent recognition that the actual number of new cases of type 1 diabetes in adults has been underreported [4]. A study from the United Kingdom indicated that the numbers of new cases are evenly distributed above and below the age of 30 [5], accentuating the statement by the American Diabetes Association, that “[t]*he precise incidence of new-onset type 1 diabetes in those over 20 years of age is unknown*” [6].

In the United States, the advent of large databases from health insurance companies that offer nationwide coverage affords an opportunity to investigate countrywide patterns of disease. In 2015, 70% of people in the United States under the age of 65 years received their healthcare through private insurance [7]. Using such a national database, we were particularly interested in the incidence of type 1 diabetes of acute onset, rather than slowly developing disease, which is referred to as latent autoimmune diabetes of adult-onset (LADA) [8]. To our knowledge, this is the first nationwide report of incidence rates of type 1 diabetes mellitus across time and place within the United States for both children and adults.

## Methods

### Subjects

In this longitudinal study, data were obtained through de-identified information retrieved from the Clinformatics® Data Mart Database (OptumInsight, Eden Prairie, MN) from a large, national, United States commercial health insurance provider. The longitudinal database contained 61 million beneficiaries who received both medical and drug coverage. The integrated enrollment, medical, and prescription claims data included information regarding both outpatient and inpatient medical services (e.g., clinic visits, rural health visits, emergency room visits, hospitalizations, etc.) with diagnoses and procedures. Pharmacy files, which included medication prescriptions, and member files, which contained general demographic information, were also obtained. The files contained information from claims submitted from January 1, 2001, through June 30, 2015.

We selected patients with newly diagnosed (incident) type 1 diabetes mellitus. Patients with type 1 diabetes were defined as those individuals with a minimum of two diagnoses indicating type 1 diabetes (ICD-9-CM codes: 250.01, 250.03, 250.11, 250.13, 250.21, 250.23, 250.31, 250.33, 250.41, 250.43, 250.51, 250.53, 250.61, 250.63, 250.71, 250.73, 250.81, 250.83, 250.91, 250.93) and at least one outpatient prescription filled for insulin (insulins 682008, 68200800). The use of administrative data (specifically, ICD-9 codes from billing data) to identify individuals with type 1 diabetes was reported to have a sensitivity of 96.7% in children under 10 years of age and of 96.9% in persons 10 years of age and older (specificity 99.8% and 99.7%, respectively) [9].

Individuals with gestational diabetes (ICD-9-CM code: 648.8×) were excluded. We also excluded individuals aged 65 years or older because the eligibility for Medicare Advantage, a government health insurance program principally for adults aged 65 years and older, varied over the time period of this study.

To detect incident (as opposed to existing) cases, we required at least 6 months (≥182 days) of insurance coverage prior to the first recorded diagnosis of type 1 diabetes. That is, we required that there be at least 6 months without any record of type 1 diabetes before the first diagnosis was reported; infants (with diagnoses codes and insulin use) were defined as having incident type 1 diabetes even if covered for less than 6 months.

For adults aged 20–64 years at diagnosis, we instituted additional procedures to distinguish type 1 from type 2 diabetes. First, we excluded individuals who used any antidiabetic drug (insulin, biguanides, dipeptidyl peptidase-4 inhibitors, incretin mimetics, meglitinides, sulfonylureas, and/or thiazolidinediones) during their first 6 months of healthcare coverage. This removed individuals being treated for type 2 diabetes at the time of study entry and individuals with pre-existing type 1 diabetes. The second criterion for adults was that insulin use after diagnosis was continuous over time. Individuals were excluded if they had received prescription refills for insulin but later discontinued use for at least 6 months even though they were still eligible for drug coverage. Third, because there may be uncertainty regarding the type of diabetes when first diagnosed, we allowed a window around the first diagnosis date during which various antidiabetic agents may be used. However, adults who used oral antidiabetic medications (biguanides, dipeptidyl peptidase-4 inhibitors, incretin mimetics, meglitinides, sulfonylureas, and/or thiazolidinediones) 6 months before or after the first type 1 diabetes diagnosis date were excluded. This primarily removed those with long-standing type 2 diabetes who were adding insulin to their regimen.

We conducted two sensitivity analyses, each using a different classification for type 1 diabetes. In the first sensitivity analysis, we mirrored the Vanderloo algorithm [10]. That is, all incident cases under the age of 10 years were selected. These cases had at least two type 1 diabetes diagnoses with an outpatient prescription for insulin filled. For individuals of 10 years of age or above, incident cases were those who used insulin only within 730 days after the date of their first diagnosis. As before, we removed existing cases during their first 6 months of healthcare coverage (to focus on incident cases) and we stipulated that, once started on insulin, they did not discontinue use.

In the second sensitivity analysis, we retained all the principal requirements for classification as described in our main analysis (above). However, we further restricted the definition of type 1 diabetes; only those individuals who had a rapid onset (at any age) were included. A rapid onset was defined as the occurrence of the first diagnosis date and the first insulin prescription filled within 6 months of each other.

### Analyses

Incidence rates were calculated from the count of incident cases (numerator) and the person-years of observation (denominator). Rates and exact Poisson 95% confidence intervals (CIs) were calculated by age group, sex, census division, and year of diagnosis. Nine divisions were defined by the Census Bureau as New England, Middle Atlantic, East North Central, West North Central, South Atlantic, East South Central, West South Central, Mountain, and Pacific [11]. Unadjusted age-specific rates were reported so that the actual numbers of persons affected (per population) are available for health services planners and for linkage in future investigations. To assess changes in incidence rates over time, negative binomial regression was used with robust standard errors, offset by the natural logarithm of person-years of observation. Because there was prior evidence of seasonal differences in incidence [12], we also plotted the percentage of incident cases by month over the entire study period (2001–2015) and differences in counts were assessed through Poisson regression. Direct standardization by age, sex, and race was used to adjust incidence rates over time, using the pooled population 2001–2015 as the standard. Alpha was set at 0.05, two-tailed. All analyses were conducted in Stata/MP version 14.2.

Calculations were performed to extrapolate to the entire United States population using Census data from the year 2015 [7]. Our cohort of 61 million privately insured Americans is a sample of the 214 million individuals covered under commercial healthcare insurers annually in the United States [7]. Therefore, we extrapolated our sample to the reference population under private healthcare coverage using age-specific rates. Next, we estimated the number of incident cases for (1) individuals who receive government (Medicaid and military) health insurance and (2) the uninsured, through direct standardization. Because the age and racial distributions of these populations differ from the commercially insured population, we first calculated age- and race-specific incidence rates of type 1 diabetes and applied these to the respective distributions for those receiving government insurance and the uninsured.

## Results

The database contained 61,795,350 individuals who had private healthcare insurance from January 2001 through June 2015. Of these people, 437,688 had two type 1 diabetes diagnoses recorded and at least one prescription filled for insulin. Overall, there were 32,476 individuals who developed type 1 diabetes in the study cohort. The incidence rate was 22.9 cases per 100,000 person-years (95% CI 22.7/100,000 to 23.2/100,000) for individuals aged 0–64 years. The mean length of health coverage for the incident cases was 5.1 years (SD, 3.2 years); the median length of health coverage was 4.3 years (interquartile range, 2.5–7.0 years).

### Incidence by age and sex

Incident cases included 13,302 youth (0–19 years) and 19,174 adults (20–64 years). The incidence rate of type 1 diabetes was greatest in youth aged 10–14 years, at 45.5 cases per 100,000 person-years (Table 1). The annual incidence rate of type 1 diabetes was 34.3 per 100,000 persons for ages 0–19 years and 18.6 per 100,000 persons for ages 20–64 years in this cohort.

**Table 1:** Incidence of type 1 diabetes mellitus and characteristics when first diagnosed by age category, 2001–2015

Age category, years^a^	Incident cases	Incidence rate^b^	Ketoacidosis within 6 months of diagnosis^c^		Emergency glucagon kit within 6 months of diagnosis^d^		Rapid onset^e^	
	n		n	%	n	%	n	%
< 5	1944	23.0 (22.0–24.1)	295	15.2%	1318	67.8%	1873	96.3%
5–9	2966	29.9 (28.9–31.0)	362	12.2%	2355	79.4%	2895	97.6%
10–14	4668	45.5 (44.2–46.8)	533	11.4%	3248	69.6%	4499	96.4%
15–19	3724	36.4 (35.3–37.6)	334	9.0%	1713	46.0%	3511	94.3%
20–24	1752	18.0 (17.2–18.9)	140	8.0%	344	19.6%	1608	91.8%
25–29	1841	16.6 (15.8–17.3)	126	6.8%	238	12.9%	1673	90.9%
30–34	1897	15.3 (14.6–16.0)	135	7.1%	201	10.6%	1693	89.2%
35–39	2042	15.9 (15.2–16.6)	164	8.0%	182	8.9%	1788	87.6%
40–44	2148	16.0 (15.3–16.7)	152	7.1%	139	6.5%	1843	85.8%
45–49	2353	17.8 (17.1–18.5)	134	5.7%	116	4.9%	2005	85.2%
50–54	2446	20.0 (19.2–20.8)	108	4.4%	117	4.8%	2063	84.3%
55–59	2424	23.4 (22.5–24.3)	83	3.4%	101	4.2%	2012	83.0%
60–64	2271	29.2 (28.0–30.4)	56	2.5%	66	2.9%	1864	82.1%
Total	32,476	22.9 (22.7–23.2)	2622	8.1%	10,138	31.2%	29,327	90.3%

^a^Age at first diagnosis

^b^Incident cases of type 1 diabetes per 100,000 person-years of healthcare coverage (95% confidence interval)

^c^Ketoacidsosis was recorded within 6 months (i.e., ±182 days) of the first diagnosis

^d^Emergency glucagon kit was filled within 6 months (i.e., ±182 days) of the first diagnosis

^e^First diagnosis of type 1 diabetes and the first insulin prescription filled were within 6 months (i.e., ±182 days)

**Fig. 1:**
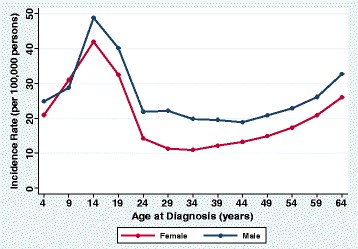
Incidence rates for type 1 diabetes by age at diagnosis and sex, United States, 2001–2015

Type 1 diabetes developed more often in males than in females (Fig. 1), with an incidence of 26.1/100,000 person-years (95% CI 25.7/100,000 to 26.5/100,000) and 19.7/100,000 person-years (95% CI 19.4/100,000 to 20.0/100,000; *P* < 0.001), respectively. The male to female incidence rate ratio was 1.32 (95% CI 1.30–1.35). The predominance in males was evident by age 10 and persisted throughout adulthood.

In the 6 month period around diagnosis, ketoacidosis occurred in a small percentage of cases, ranging from 2.5% in those aged 60–64 years to 15.2% in those aged under 5 years (Table 1). Prescriptions for emergency glucagon kits were filled most often during childhood (e.g., 79.4% in children aged 5–9 years at diagnosis) and less often in adults (e.g., 4.8% for those aged 50–54 years). For most children and adults, the first insulin prescription was filled within 6 months of the first diagnosis (>90% in youth; > 80% in older adults).

### Incidence over time

**Fig. 2:**
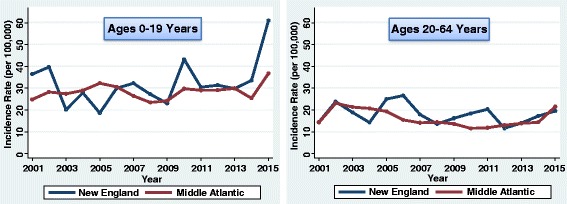
Incidence of type 1 diabetes in Northeastern United States by year

**Fig. 3:**
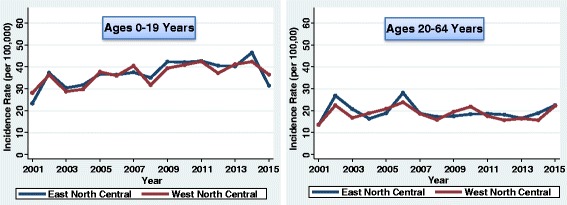
Incidence of type 1 diabetes in Midwestern United States by year

**Fig. 4:**
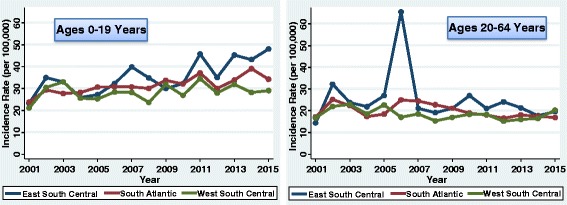
Incidence of type 1 diabetes in Southern United States by year

**Fig. 5:**
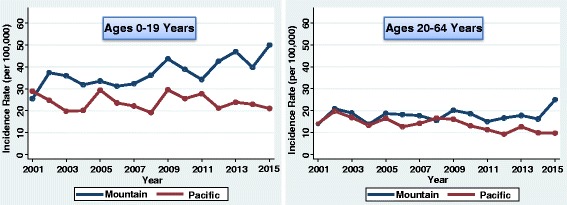
Incidence of type 1 diabetes in Western United States by year

The incidence of type 1 diabetes in youth (0–19 years) increased by 1.9% each year from 2001 to 2015 (95% CI 1.1–2.7%; *P* < 0.001). However, there was variation by division of the country (Figs. 2, 3, 4, and 5). The greatest increase was in the East South Central division (3.8%/year; 95% CI 2.0–5.6%; *P* < 0.001). The next greatest increase occurred in the Mountain division (3.1%/year; 95% CI 1.6–4.6%; *P* < 0.001). There were also increases in the East North Central (2.7%/year; 95% CI 0.7–4.9%; *P* = 0.010), South Atlantic (2.4%/year; 95% CI 1.3–3.4%; *P* < 0.001), and West North Central divisions (2.4%/year; 95% CI 1.2–3.6%; *P* < 0.001). There were no significant linear increases in incidence in the Middle Atlantic (*P* = 0.441), New England (*P* = 0.370), West South Central (*P* = 0.229), or Pacific divisions (*P* = 0.558). Although there was no significant linear increase in New England youth, the rates fluctuated considerably over time. CIs for the plotted rates are given in Additional file 1: Tables S1–S4.

For adult-onset type 1 diabetes, there was a decrease in the incidence from 2001 to 2015 (−1.3%/year; 95% CI −2.3% to −0.4%; *P* = 0.007). This decrease was driven by the Pacific division (−3.7%/year; 95% CI −5.3% to −1.9%; *P* < 0.001). The other regions did not experience a significant linear trend in incidence rates in adults. However, there was a spike in incidence in the year 2006 for adults in the East South Central region (Fig. 4); the incidence was elevated in both men (68.8 per 100,000) and women (62.1 per 100,000).

After adjustment for age, sex, and race, the increase in the incidence rates of type 1 diabetes from 2001 to 2015 in youth remained. The adjusted incidence rates increased by 0.9% each year in youth (*P* = 0.009). In adults aged 20–64 years, the reduction in incidence rates over time remained after adjustment. There was a 1.9% reduction in incidence rates with adjustment (*P* = 0.026).

**Fig. 6:**
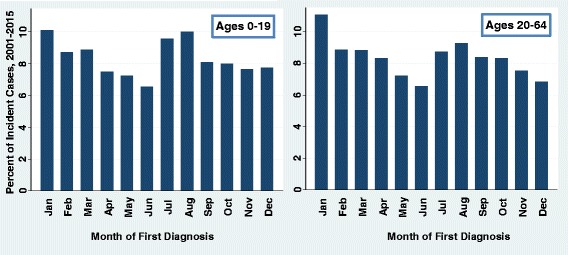
Month of first diagnosis for type 1 diabetes in the United States, 2001–2015

Variation in the percentages of incident cases by month of first diagnosis is shown in Fig. 6. The greatest percentages of cases were diagnosed in January for both youth and adults. There were also increased proportions diagnosed in July and August. For youth, counts of incident cases were not significantly different in January versus July (*P* = 0.177) or August (*P* = 0.847), but were different from all the other months (*P* < 0.001). In adults, the number of incident cases in January was significantly greater than any of the other months (*P* < 0.001 for all other months). The monthly patterns appeared somewhat similar in youth and adults, although the percentage of incident cases in July and August were more elevated in youth.

### Sensitivity analyses

Using the Vanderloo algorithm yielded slightly higher incidence rates of type 1 diabetes, particularly in the older age categories (Table 2). The overall incidence was 26.1 cases per 100,000 person-years (95% CI 25.8/100,000 to 26.4/100,000). The more restrictive classification of limiting cases to only those with a rapid onset yielded a slightly lower incidence of type 1 diabetes (20.7 cases/100,000 person-years; 95% CI 20.4/100,000 to 20.9/100,000); the age-specific rates are shown in Table 2.

**Table 2:** Sensitivity analyses for the incidence of type 1 diabetes mellitus in the United States by age category, 2001–2015

	Vanderloo algorithm^a^			Rapid onset^b^		
Age category, years^c^	N, incident cases	Incidence rate^d^	95% Confidence intervals	N, incident cases	Incidence rate^d^	95% Confidence intervals
< 5	1944	23.0	22.0–24.1	1873	22.2	21.2–23.2
5–9	2966	29.9	28.9–31.0	2895	29.2	28.2–30.3
10–14	4466	43.6	42.3–44.8	4499	43.9	42.6–45.2
15–19	3399	33.3	32.1–34.4	3511	34.4	33.2–35.5
20–24	1746	17.9	17.1–18.8	1608	16.5	15.7–17.3
25–29	1943	17.5	16.7–18.3	1673	15.0	14.3–15.8
30–34	2095	16.9	16.2–17.6	1693	13.7	13.0–14.3
35–39	2418	18.8	18.0–19.5	1788	13.9	13.2–14.5
40–44	2741	20.4	19.7–21.2	1843	13.7	13.1–14.4
45–49	3090	23.4	22.5–24.2	2005	15.2	14.5–15.8
50–54	3481	28.4	27.5–29.4	2063	16.9	16.1–17.6
55–59	3436	33.1	32.0–34.3	2012	19.4	18.6–20.3
60–64	3356	43.2	41.7–44.7	1864	24.0	22.9–25.1
Total	37,081	26.1	25.8–26.4	29,327	20.7	20.4–20.9

^a^All incident cases < 10 years of age; for those ≥ 10 years, insulin only within 730 days from

diagnosis

^b^First diagnosis of type 1 diabetes and the first insulin prescription filled were within 6 months

^c^Age at first diagnosis

^d^Incident cases of type 1 diabetes per 100,000 person-years of healthcare coverage

### Extrapolation to the entire population

Overall, for individuals aged 0–64 years, the number of annual incident cases of type 1 diabetes in the United States was estimated to be 64,000, regardless of insurance type. For youth, the number of incident type 1 diabetes cases was estimated to be 27,000 annually (15,000 with commercial health insurance, 11,000 with government health insurance, 1000 uninsured). For adults (20–64 years of age), we estimated 37,000 incident cases of type 1 diabetes annually in the United States (24,000 with commercial health insurance, 8000 with government insurance, and 5000 uninsured).

## Discussion

There are more new cases of type 1 diabetes occurring each year in the United States than previously recognized. Overall, the number of individuals (0–64 years of age) who develop type 1 diabetes annually is approximately 64,000 across the entire country. More incident cases of type 1 diabetes occur in adults than in youth each year, which is similar to the findings from the United Kingdom [5]. While the incidence rate is greater in youth, adulthood extends for a longer time. Thus, this phenomenon might have been predicted by population dynamics while underappreciated in everyday practice. The SEARCH for Diabetes in Youth Study estimated that there were 17,900 youth (aged < 20 years) who developed type 1 diabetes in 2011–2012 in the United States [13]. Earlier estimates were 13,000 new cases on an annual basis in youth [1]. Our study, using data from the entire United States, suggests that these figures are likely underestimates. A possible reason for the difference is that earlier estimates were based on data from only five states, while our analysis included data from all 50 states.

Data from Sweden indicate that the incidence of type 1 diabetes is bimodal [14], wherein two spikes in incidence are observed, one at ages 0–9 years and another occurring at 50–80 years of age [14]. In our database, there was a spike in incidence at ages 10–14 years, followed by a decline, and then an upward trend starting at age 40. Similar findings were reported from the population-based registry in Turin, Italy [15], wherein a rise in incidence at ages 10–14, followed by a decline and a slow increase from age 30 onwards was observed. Similar to our study, the authors found the same pattern of a male predominance from age 10 onwards. Others have also observed such male predominance; a recent review found that 44 of 54 studies reported male predominance in adolescents and adults [16]. Populations with higher incidences of type 1 diabetes overall tend to have greater male predominance [17]. Studies of sex differences in hormonal fluctuations at the time of adrenarche in genetically predisposed individuals could perhaps provide clues to the underlying pathogenesis of this disease.

We found that incidence rates of type 1 diabetes in youth significantly increased with time. The SEARCH study reported an annual increase of 2.7% from 2002 to 2009 in non-Hispanic white youth [18] and an adjusted annual increase of 1.8% in all youth between the periods 2002–2003 and 2011–2012 [13]. We found an annual increase of 1.9% from 2001 to 2015 when measured in all youth. Of note, the East South Central, Mountain, Midwest, and South Atlantic regions demonstrated an increase of 2.4% to 3.8% annually. Variations in the incidence of childhood type 1 diabetes have also been observed in Europe, with increases ranging from 0.4% to 7.8% across different regions (the exception being Catalonia) from 1999 to 2008 [19].

However, we found no increase over time in the incidence of adult-onset cases. With the data available, the underlying reasons for this cannot be directly determined. Although both youth- and adult-onset cases are described as autoimmune in nature, etiologic determinants are still under investigation in children and have not been thoroughly studied in adults. Of note, there was one irregularity, wherein the data indicated a spike in incidence in 2006 in adults of the East South Central region. This could have been due to differences in diagnosis at that time, factors related to the particular patients in the region, a recording error, or true incident cases. Clusters of cases have been anecdotally reported in specific areas [20], but further investigation would be needed to determine relevant factors.

We found seasonal differences in incidence rates, with somewhat similar patterns in youth and adults. Seasonal variation in diagnosis of type 1 diabetes has been widely reported in the literature and, in general, show peaks in the winter and troughs in late spring to summer in the northern hemisphere [12]. Because the etiology is multifactorial [21], the significance of such seasonal patterns is unclear. One report indicated a median of 25 days (range 2–315 days) from symptom onset to diagnosis in children [22], but the time period from pathogenesis to symptom onset is speculative.

Our results provide a clue that the etiologic factors contributing to pediatric type 1 diabetes differ from those of adult-onset disease. Incidence rates increased over time in youth-onset cases while these rates decreased in adults, suggesting that some precipitating factors may be different in early- versus later-onset disease. Moreover, regional variations in incidence suggest that the etiologic contributors are differentially distributed across the country.

There are limitations to our investigation. Data regarding C-peptide and glutamic acid decarboxylase autoantibodies were only available on a small fraction of cases. Therefore, the diagnosis was determined through ICD-9-CM diagnosis codes and medication use. Validation studies indicate that the use of diagnosis codes and medications yield a sensitivity of 98.6% for type 1 diabetes [10] and, for diagnosis codes alone, a positive predictive value of 97.0% for type 1 diabetes [23]. The determination of adult-onset type 1 diabetes was more difficult because, in practice, it may be mistakenly diagnosed as type 2 diabetes. Therefore, we instituted additional measures and conducted sensitivity analyses that restricted the number of adults classified as having type 1 diabetes. Although adults newly diagnosed with type 2 diabetes are usually treated with oral agents and lifestyle measures rather than insulin exclusively, it is possible that adults with extreme glycemia elevations and/or contraindications to oral agents would be treated initially with insulin and would continue to use insulin exclusively. It is also possible that our definition in adults may have been too restrictive, because some individuals with type 1 diabetes may use metformin [24], although this generally occurs with established cases of type 1 diabetes and not with newly onset disease, as assessed herein. Finally, we used standard diagnosis codes as currently available. For adult-onset autoimmune diabetes mellitus, there have been recent discussions regarding reclassification of a subset of adults in which β-cell failure occurs slowly (LADA) [8, 25]. There is considerable controversy regarding the use of the term LADA, as well as suggestions for improving the current classification of diabetes based on β-cell commonalities across all presentations of the disease [26, 27]. Population-based longitudinal studies will be necessary to determine what fraction of the adult-onset autoimmune cases this constitutes, although some investigators report LADA to be much more prevalent than acute-onset type 1 diabetes [28, 29]. In our study, the focus was on acute-onset cases; we did not capture LADA cases, although this is of interest for future studies.

One of the strengths of our study is the use of integrated medical files to identify cases. In the past, registries or lists from single sources were used, with capture-recapture methods to extend ascertainment [4, 18, 30, 31]. The relational database used contained all the different locations in which patients received services within their health insurance plan, which included clinician visits (primary care physicians, specialists, nurse practitioners, etc.), home health visits, rural health visits, urgent care visits, ambulance services, hospitalizations (acute care, long-term, rehabilitation), skilled nursing facilities, mental health services, as well as all prescriptions with relevant dates. The use of such large national databases may provide exceptional and complimentary information to studies that utilize existing registries.

## Conclusions

More new cases of type 1 diabetes occur each year in the United States than previously recognized. Incident cases occurred more often in adults than youth, even though incidence rates were greater in youth because of the longer time period encompassing adulthood. From 2001 to 2015, incidence rates of type 1 diabetes increased in youth, but not uniformly throughout the country. However, incidence rates decreased over time in adults. The increase in incidence rates in youth, but not adults, suggests that the precipitating factors of youth-adult disease may differ from those of adult-onset disease. Fluctuations in the incidence of type 1 diabetes may provide clues to etiology and facilitate the planning of appropriate health services.

## Additional file

Additional file 1:
**Tables S1** and **S2.** Incidence rates of type 1 diabetes in patients, ages 0-19 years. **Tables S3** and **S4**. Incidence rates of type 1 diabetes in patients, ages 20-64 years. (ZIP 26.3 kb)

## Abbreviations

CI: Confidence interval
ICD-9-CM: International Classification of Diseases, Ninth Revision, Clinical Modification
